# EPYSQLI (SB12; Biosimilar to Reference Eculizumab) in Asian and Non‐Asian Patients With Paroxysmal Nocturnal Hemoglobinuria: Subgroup Analysis of a Global Phase III Randomized Controlled Trial

**DOI:** 10.1002/jha2.70020

**Published:** 2025-03-21

**Authors:** Jun Ho Jang, Ciprian Tomuleasa, Hanna Oliynyk, Theerin Lanamtieng, Jihye Park, Younsoo Kim, Jinah Jung, Paola Russo, Soo Min Lim, Régis Peffaultde Latour

**Affiliations:** ^1^ Department of Hematology‐Oncology, Samsung Medical Center, Sungkyunkwan University School of Medicine Seoul Republic of Korea; ^2^ Department of Hematology Iuliu Hatieganu University of Medicine and Pharmacy Cluj‐Napoca Romania; ^3^ Department of Hematology Ion Chiricuta Clinical Cancer Center Cluj‐Napoca Romania; ^4^ Medfuture Research Center for Advanced Medicine Iuliu Hatieganu University of Medicine and Pharmacy Cluj‐Napoca Romania; ^5^ Department of Hematology Vinnytsia M I Pyrohov Regional Clinical Hospital Vinnytsia Ukraine; ^6^ Department of Internal Medicine Division of Hematology Srinagarind Hospital, Faculty of Medicine, Khon Kaen, University Khon Kaen Thailand; ^7^ Samsung Bioepis Co., Ltd. Incheon Republic of Korea; ^8^ Department of Internal Medicine Sultanah Aminah Hospital Johor Bahru Malaysia; ^9^ Hematology and Transplant Unit French Reference Center for Aplastic Anemia and Paroxysmal Nocturnal Hemoglobinuria Assistance Publique‐Hôpitaux de Paris, Université Paris Cité Paris France

**Keywords:** Asian, clinical trial, paroxysmal nocturnal hemoglobinuria, PNH, SB12, subgroup analysis

## Abstract

**Introduction:**

SB12 demonstrated equivalence to reference eculizumab (ECU) in complement inhibitor‐naïve patients with paroxysmal nocturnal hemoglobinuria (PNH) in the previous randomized, double‐blind, multi‐national, crossover, Phase III study.

**Methods:**

The scope of this post‐hoc study was subgroup analysis by race to compare the efficacy and safety of SB12 and ECU in PNH patients in the Asian and Non‐Asian subgroups of the Phase III study.

**Results:**

Results including lactate dehydrogenase (LDH), number of units of packed red blood cells and safety as primary and secondary endpoints demonstrated comparable efficacy and safety of SB12 and ECU in Asian and Non‐Asian PNH patients, in line with the study results in the overall population. In addition, transfusion avoidance (68.1% for SB12 vs. 72.9% for ECU, *p*‐value of 0.4492) and hemoglobin stabilization (SB12–ECU: 6.3%, 95% confidence interval [CI] [‐21.5, 34.1] and SB12–ECU: 2.5%, 95% CI [‐24.8, 29.8] using stringent criteria) as post‐hoc endpoints were not substantially different between SB12 and ECU treatment groups in the overall population as well as in Asians and Non‐Asians.

**Conclusion:**

In conclusion, this subgroup analysis by race (Asians and Non‐Asians) supports comparable efficacy and safety between SB12 and reference eculizumab in global PNH patients including no difference in transfusion avoidance effect.

## 1 Introduction

Paroxysmal nocturnal hemoglobinuria (PNH) is a rare, acquired clonal hematopoietic stem cell disorder characterized by hemolytic anemia, bone marrow failure, and thrombosis, which leads to significant morbidity and mortality if untreated [1, 2, 3, 4, 5]. The disorder is caused by a somatic mutation in the phosphatidylinositol glycan class‐A gene, leading to a deficiency of glycosylphosphatidylinositol (GPI)‐anchored proteins, CD55 and CD59, resulting in uncontrolled complement activation and intravascular hemolysis [3]. Paroxysms resulting in brisk hemolysis are triggered by surgery, infection, or inflammation [3].

Eculizumab, a humanized monoclonal antibody targeting complement C5, has transformed the treatment of PNH by preventing complement‐mediated hemolysis and improving clinical outcomes, including eliminating or reducing thrombosis risk, blood transfusion requirements, and improving anemia, quality of life, and survival [3, 6, 7]. EPYSQLI (SB12; Samsung Bioepis), a biosimilar to the eculizumab reference product (ECU; Soliris, Alexion Pharmaceuticals), was first approved by the European Medicines Agency (EMA) in May 2023 to treat patients with PNH and signs of hemolysis, in line with ECU [8, 9]. Following extensive analytical characterization [10], a Phase I study (NCT03722329) showed equivalent pharmacokinetics (PK) and comparable pharmacodynamics (PD), safety, and immunogenicity profiles between SB12 and ECU [11, 12]. A randomized, double‐blind, multi‐national, cross‐over, Phase III study (NCT04058158) demonstrated equivalent clinical efficacy and comparable safety, PK, PD, and immunogenicity between SB12 and ECU in complement‐inhibitor naïve PNH patients [13].

Here, we present a post‐hoc efficacy and safety analysis of the Phase III study by subgroup in Asians and Non‐Asians to analyze the comparability of SB12 and ECU results by race, relative to the results obtained in the overall population.

## 2 Patients and Methods

This randomized, double‐blind, multi‐national, cross‐over, Phase III study (NCT04058158) was conducted at 24 study centers in eight countries (in alphabetical order, India, Malaysia, Mexico, Republic of Korea, Romania, Taiwan, Thailand, Ukraine) between August 2019 and October 2021. Study design, population, outcomes, and statistical analyses were previously reported [13].

Fifty adults with PNH, ≥1.5 upper limit of normal (ULN) range of lactate dehydrogenase (LDH) and complement inhibitor‐naïve were randomized (1:1) to treatment sequence I (TS1: SB12–ECU, *n* = 25) or II (TS2: ECU–SB12, *n* = 25), to receive 600 mg of SB12 or ECU intravenously every week for first 4 weeks and 900 mg for the fifth week, followed by 900 mg every 2 weeks thereafter. Upon completing Period 1 at Week 26, patients were switched to ECU or SB12, respectively, and treated until completing Period 2 at Week 50.

The Phase III primary efficacy endpoints included the LDH level at Week 26 and the time‐adjusted area under the effect curve (AUEC) of LDH from Week 14 to Week 26 and Week 40 to Week 52. Equivalence was declared for LDH level at Week 26 if the two‐sided 95% confidence interval (CI) of the mean difference between SB12 and ECU fell within the pre‐defined equivalence margin of [−1.2 × ULN, 1.2 × ULN] = [−337.2, 337.2], where ULN = 281 U/L. In addition, equivalence was declared for time‐adjusted AUEC of LDH if the two‐sided 90% CI of the ratio of geometric means between SB12 and ECU fell within the pre‐defined equivalence margin of [0.77, 1.29]. Secondary efficacy endpoints included the LDH profile over time and the number of transfused packed red blood cell (pRBC) units as previously described [13]. Overall, post‐hoc analysis in Asian and Non‐Asian (based on race) patients who received either ECU or SB12 during the study period was conducted for the aforementioned Phase III efficacy endpoints.

The Phase III safety endpoints included the incidence of adverse events (AEs), treatment‐emergent AEs (TEAEs), serious AEs (SAEs), and AEs of special interest (AESIs: infection‐related AEs and infusion‐related reactions). This safety subgroup analysis was conducted in pooled patients treated with SB12 or ECU in either Periods 1 or 2 by Asian and Non‐Asian subgroups (by race).

### 2.1 Additional (Post‐hoc) Efficacy Analysis Methods

#### 2.1.1 Transfusion Avoidance

Transfusion avoidance was achieved by subjects who never received pRBC transfusion during the treatment period.

For the overall population, differences in the proportion of subjects achieving transfusion avoidance were calculated using a binomial model (implemented via SAS procedure GENMOD) with TS, period, and treatment group as effects, transfusion avoidance as a response, and repeated subject effect for the Modified Full Analysis Set (M‐FAS). The response was assumed to follow a binomial distribution with the identity link function. The difference in proportions of subjects achieving transfusion avoidance between SB12 and ECU and corresponding 95% CI were provided. For subgroup analysis by race (Asians and Non‐Asians), the number of subjects and percentage of subjects with transfusion avoidance were summarized by TS, study period, and race (Asian/Non‐Asian).

#### 2.1.2 Hemoglobin Stabilization

Given the lack of standardized criteria for defining hemoglobin stabilization and the post‐hoc nature of this analysis, two criteria were defined for comparative analysis:

- Criterion 1 [14]: Hemoglobin stabilization was defined as a subject who never received pRBC transfusion during Study Period 1 and whose hemoglobin did not decrease by >10 g/L (post‐baseline value—baseline value ≥ ‐10 g/L) from baseline during Study Period 1. This criterion represents the avoidance of a >10 g/L decrease in hemoglobin levels without pRBC transfusions.
- Criterion 2 [15, 16]: Hemoglobin stabilization was defined as a subject who never received pRBC transfusion during Study Period 1 and whose hemoglobin did not decrease by ≥20 g/L (post‐baseline value—baseline value > ‐20 g/L) from baseline during Study Period 1. This criterion represents the avoidance of a ≥ 20 g/L decrease in hemoglobin levels without pRBC transfusions.

The difference in the proportion of subjects achieving hemoglobin stabilization was calculated using the Cochran‐Mantel‐Haenszel (CMH) test, and the corresponding 95% Wald CIs were presented for the M‐FAS. The analysis of the difference in the proportion of subjects achieving hemoglobin stabilization was also performed by race (Asian/Non‐Asian) and for the overall population.

## 3 Results

In total, 50 patients were enrolled in the Phase III study and Asian patients were 27 (54.0%) among those patients. Overall, 15 (60%) Asian patients and 10 (40%) Non‐Asian patients were randomized to TS1 (SB12–ECU, *n* = 25), and 12 (48%) Asian patients and 13 (52%) Non‐Asian patients were randomized to TS2 (ECU–SB12, *n* = 25).

Baseline demographic and disease characteristics were comparable between the two TSs, with no significant differences between the TSs by race (Asian and Non‐Asian patients) (Tables S1 and S2).

### 3.1 Efficacy

#### 3.1.1 Phase III Primary Efficacy Endpoints by Subgroup

The 95% CI of the mean difference in LDH level at Week 26 between SB12 and ECU in Asian (SB12–ECU: ‐12.02, 95% CI [−126.87, 102.83]) and Non‐Asian patients (SB12–ECU: 76.12, 95% CI [−56.75, 208.99]) fell within the pre‐defined equivalence margins (Table 1). The 90% CI of the ratio of time‑adjusted AUEC of LDH between SB12 and ECU in Asian patients (SB12/ECU: 1.01, 90% CI [0.92, 1.10]) fell within the pre‑defined equivalence margin and was also comparable in Non‐Asian patients (SB12/ECU: 1.15, 90% CI [0.90, 1.46]). (Table 1).

**TABLE 1:** Post‐hoc subgroup analysis of Phase III primary efficacy endpoints (lactate dehydrogenase [LDH] at week 26 and time‐adjusted area under the effect curve [AUEC] of LDH) by race.

Lactate Dehydrogenase (U/L) at Week 26					
	SB12		ECU		Mean difference (SB12–ECU)
**Subgroup**	***n***	**LSM**	***n***	**LSM**	**Estimate (95% CI)**
Asian	13	269.71	10	281.73	−12.02 (‐126.87, 102.83)
Non‐Asian	10	309.84	13	233.72	76.12 (‐56.75, 208.99)

Time‐adjusted AUEC (U/L) of LDH					
	SB12		ECU		Ratio (SB12/ECU)
**Subgroup**	***n***	**Geometric LSM**	***n***	**Geometric LSM**	**Estimate (90% CI)**
Asian	18	280.58	18	278.52	1.01 (0.92, 1.10)
Non‐Asian	20	286.74	20	249.87	1.15 (0.90, 1.46)

*Note*: AUEC = area under the effect curve; CI = confidence interval; ECU = reference eculizumab; LDH = Lactate dehydrogenase; LSM = least squares mean; *n* = number of patients in each subgroup.

#### 3.1.2 Phase III Secondary Efficacy Endpoints by Subgroup

**FIGURE 1:**
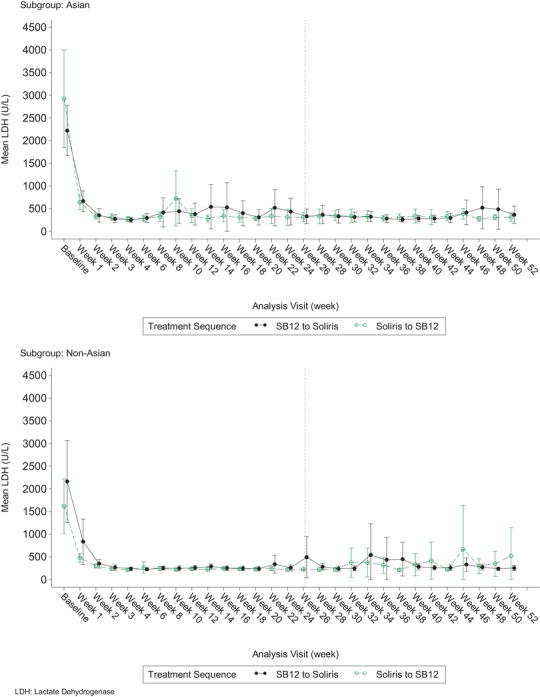
Plot of mean 95% confidence interval of lactate dehydrogenase (LDH) profile by race.

The overall level of LDH (U/L) was also comparable between TSs by race with Asian and Non‐Asian patients throughout the study period. As observed in the overall population [13], LDH levels decreased significantly after the first dose in both Asians and Non‐Asians, and the mean LDH stayed below 2 times the ULN of LDH from week 3 onwards (Figure 1).

Among baseline disease characteristics, the mean pRBC units transfused in 12 months prior to screening were 6.4 U in planned TS1 (SB12–ECU) and 3.8 U in planned TS2 (ECU to SB12) [13]. A slightly higher number of pRBC units were transfused into Asians (8.3 U in planned TS1 and 5.0 U in planned TS2) compared to Non‐Asians (3.4 U in planned TS1 and 2.6 U in planned TS2) prior to screening (Table S2).

After study treatment initiation, the mean units of pRBCs transfused were decreased in both planned TSs analyzed by race (Asian, Non‐Asian), similarly to the overall population [13]. In particular, during the overall study duration, the mean number of units of pRBCs was comparable between TSs in Asians (4.6 U in planned TS1 and 4.5 U in planned TS2) and Non‐Asians (0.5 U in planned TS1 and 1.0 U in planned TS2). (Table 2) By Asian and Non‐Asian subgroup analysis, there was no statistically significant difference in the median number of units of pRBCs transfused between the planned TSs throughout the study duration and by study period (Table 2), as it was previously observed in the overall population [13].

**TABLE 2:** Post‐hoc subgroup analysis of the number of units of packed red blood cells (pRBCs) transfused throughout the study duration by race.

Subgroup	Statistics	TS1 (SB12–ECU) *Units of pRBCs Transfused*	TS2 (ECU–SB12) *Units of pRBCs Transfused*	Total *Units of pRBCs Transfused*
**Asians**				
Study Overall	*n*	14	12	26
	Mean ± SD	4.6 ± 10.33	4.5 ± 6.23	4.5 ± 8.52
	Median (Min, Max)	0.0 (0, 39)	2.5 (0, 22)	2.0 (0, 39)
	*p*‐value	0.3689		
Study Period 1	*N*	14	12	26
	Mean ± SD	1.9 ± 4.79	1.8 ± 2.77	1.8 ± 3.91
	Median (Min, Max)	0.0 (0, 18)	0.0 (0, 8)	0.0 (0, 18)
	*p*‐value	0.5610		
Study Period 2	*N*	13	10	23
	Mean ± SD	2.0 ± 5.31	2.0 ± 3.77	2.0 ± 4.60
	Median (Min, Max)	0.0 (0, 19)	0.0 (0, 12)	0.0 (0, 19)
	*p*‐value	0.5785		
**Non‐Asians**				
Study Overall	*n*	10	13	23
	Mean ± SD	0.5 ± 0.85	1.0 ± 2.27	0.8 ± 1.78
	Median (Min, Max)	0.0 (0, 2)	0.0 (0, 8)	0.0 (0, 8)
	*p*‐value	0.8800		
Study Period 1	*n*	10	13	23
	Mean ± SD	0.0 ± 0.00	0.2 ± 0.38	0.1 ± 0.29
	Median (Min, Max)	0.0 (0, 0)	0.0 (0, 1)	0.0 (0, 1)
	*p*‐value	0.4862		
Study Period 2	*n*	10	13	23
	Mean ± SD	0.0 ± 0.00	0.2 ± 0.60	0.1 ± 0.46
	Median (Min, Max)	0.0 (0, 0)	0.0 (0, 2)	0.0 (0, 2)
	*p*‐value	0.4862		

*Note: N* total number of subjects in the Modified Full Analysis Set.

*p*‐Values were based on the Wilcoxon rank sum test for the testing of the treatment sequence difference.

#### 3.1.3 Additional (Post‐hoc) Efficacy Analysis

In the 12 months prior to screening, a total of 30 (60%) patients [16 (64%) in planned TS1 and 14 (54%) in planned TS2, respectively] received at least one unit of pRBCs [13]. During the overall study period, transfusion avoidance was observed in 68.1% of patients receiving SB12 vs 72.9% of patients receiving ECU, with a difference of ‐5.3% (95% CI, [‐18.9%, 8.4%], *p*‐value 0.4492). This difference was not statistically significant between the two treatment groups. Due to the small sample size, transfusion avoidance by race subgroup (Asians and Non‐Asians) was analyzed by descriptive statistics and by TS by SB12 or ECU treatment for the overall population. In the Asian subgroup, a numerical difference was noted in TS2 (five patients [41.7%] in Period 1 and six [60.0%] in Period 2) compared to TS1 (eight patients [57.1%] in Period 1 and 10 [76.9%] in Period 2). In Non‐Asian, transfusion avoidance was comparable between TS1 (seven patients [70.0%] in Period 1 and 10 [100.0%] in Period 2) and TS2 (10 patients [76.9%] in Period 1 and 11 [84.6%] in Period 2). However, the clinical significance of transfusion avoidance by race subgroup is limited by the very small number of subjects. Overall, transfusion avoidance results in the whole study population and by race subgroup appeared consistent with historical data on eculizumab [8, 9], with no clinically significant differences.

Hemoglobin stabilization during Period 1 (i.e., in subjects treated with SB12 or ECU before switching) was analyzed by both less stringent (Criterion 1) and more stringent (Criterion 2) criteria, given the lack of standardization [14, 15, 16]. (Table 3) By Criterion 1 [14], the difference in hemoglobin stabilization (SB12–ECU) was 6.3% (95% CI, ‐21.5%, 34.1%) in the overall population, 25.0% (95% CI, ‐10.9%, 60.9%) in Asians, and ‐6.9% (95% CI, ‐43.4%, 29.6%) in Non‐Asians. By Criterion 2 [15, 16], the difference in hemoglobin stabilization (SB12 – ECU) was 2.5% (95% CI, ‐24.8%, 29.8%) in the overall population, 15.5% (95% CI, ‐22.6%, 53.6%) in Asians, and ‐6.9% (95% CI, ‐43.4%, 29.6%) in Non‐Asians. Overall, no substantial differences in hemoglobin stabilization were identified between SB12 and ECU treatment groups in the overall population as well as in Asians and Non‐Asians.

**TABLE 3:** Post‐hoc analysis of the difference in hemoglobin stabilization in the overall population and by race during Period 1.

		(Post‐hoc) Patients with Hemoglobin Stabilization			
	Treatment	Criterion 1	Difference (SB12–ECU)	Criterion 2	Difference (SB12–ECU)
		*n* (%)	Estimate (95% CI)	*n* (%)	Estimate (95% CI)
**Overall population**	SB12 (*N* = 24)	14 (58.3)	6.3% (‐21.5%, 34.1%)	15 (62.5)	2.5% (‐24.8%, 29.8%)
	ECU (*N* = 25)	13 (52.0)		15 (60.0)	
**Asian**	SB12 (*N* = 14)	7 (50.0)	25.0% (‐10.9%, 60.9%)	8 (57.1)	15.5% (‐22.6%, 53.6%)
	ECU (*N* = 12)	3 (25.0)		5 (41.7)	
**Non‐Asian**	SB12 (*N* = 10)	7 (70.0)	−6.9% (‐43.4%, 29.6%)	7 (70.0)	−6.9% (‐43.4%, 29.6%)
	ECU (*N* = 13)	10 (76.9)		10 (76.9)	

*Note*: CI: Confidence Interval. Hemoglobin stabilization of Criterion 1 is defined as subjects who never received packed red blood cells (pRBCs) transfusion during Study Period 1 and whose hemoglobin did not decrease more than 10 g/L from baseline during Study Period 1 [14].

Hemoglobin stabilization of Criterion 2 is defined as subjects who never received packed red blood cells (pRBCs) transfusion during Study Period 1 and whose hemoglobin did not decrease of equal to or more than 20 g/L from baseline during Study Period 1 [15, 16].

Difference and 95% CI were estimated by the Cochran‐Mantel‐Haenszel (CMH) test.

### 3.2 Safety

Twenty‐six (53%) Asians and 23 (47%) Non‐Asians received at least one dose of either SB12 or ECU and were analyzed (Table 4). The TEAEs occurred in 22 (84.6%) Asians and 20 (87.0%) Non‐Asians, with similar incidence and safety profiles between SB12 and ECU treatments. The majority of TEAEs were mild to moderate and transient in both subgroups. While numerical differences were noted in terms of treatment‐related TEAEs between Asian and Non‐Asian patients (eight [30.8%] patients in Asians and three [13.0%] patients in Non‐Asians), this was attributed to the AESIs of infusion‐related reactions (three [11.5%] patients in Asians vs. none in Non‐Asians) and a single case of systemic infection (one [3.8%] patient in Asians vs. none in Non‐Asians) in particular, since the causality of all reported AESIs was evaluated as treatment‐related in the Phase III study (Table 4). There were no reported meningococcal infections (Table 4). Results by race subgroup were consistent with those previously reported in the overall population [13] and with the known safety profile of eculizumab [8, 9]. No patient developed anti‐drug antibodies during the overall study period [13].

**TABLE 4:** Post‐hoc subgroup analysis of treatment adverse events and treatment‐emergent adverse events by race.

	Asian^a^			Non‐Asian		
	SB12 (*N* = 24)	ECU (*N* = 25)	Total (*N* = 26)	SB12 (*N* = 23)	ECU (*N* = 22)	Total (*N* = 23)
	Person‐years = 11.7	Person‐years = 9.9	Person‐years = 21.6	Person‐years = 11.7	Person‐years = 9.8	Person‐years = 21.5
	*n* (%), E	*n* (%), E	*n* (%), E	*n* (%), E	*n* (%), E	*n* (%), E
**TEAEs**	18 (75.0), 58	19 (76.0), 55	22 (84.6), 113	16 (69.6), 61	13 (59.1), 44	20 (87.0), 105
Related	1 (4.2), 2	7 (28.0), 12	8 (30.8), 14	2 (8.7), 3	1 (4.5), 3	3 (13.0), 6
Not related	17 (70.8), 56	12 (48.0), 43	14 (53.8), 99	14 (60.9), 58	12 (54.5), 41	17 (73.9), 99
**TEAE leading to IP discontinuation**	0 (0.0), 0	1 (4.0), 1	1 (3.8), 1	0 (0.0), 0	0 (0.0), 0	0 (0.0), 0
**TEAE leading to death**	0 (0.0), 0	1(4.0), 1	1 (3.8), 1	0 (0.0), 0	0 (0.0), 0	0 (0.0), 0
**Adverse Events of Special Interest**	0 (0.0), 0	4, (16.0), 5	4 (15.4), 5	0 (0.0), 0	0 (0.0), 0	0 (0.0), 0
Meningococcal infection	0 (0.0), 0	0 (0.0), 0	0 (0.0), 0	0 (0.0), 0	0 (0.0), 0	0 (0.0), 0
Other systemic infection	0 (0.0), 0	1 (4.0), 1	1 (3.8), 1	0 (0.0), 0	0 (0.0), 0	0 (0.0), 0
Infusion‐related reaction	0 (0.0), 0	3 (12.0), 4	3 (11.5), 4	0 (0.0), 0	0 (0.0), 0	0 (0.0), 0
**Serious TEAEs**	2 (8.3), 2	2 (8.0), 3	4 (15.4), 5	1 (4.3), 1	0 (0.0), 0	1 (4.3), 1
Related	0 (0.0), 0	2 (8.0), 2	2 (7.7), 2	0 (0.0), 0	0 (0.0), 0	0 (0.0), 0
Not related	2 (8.3), 2	0 (0.0), 0	2 (7.7), 3	1 (4.3),1	0 (0.0), 0	1 (4.3), 1

*Note*: AE = adverse event; E = frequency of TEAEs; IP = investigational product; n = number of patients with event; TEAEs = treatment‐emergent adverse events; *N* for SB12 represents the total number of pooled patients who have been treated with SB12 in either Period 1 or 2; *N* for ECU represents the total number of pooled patients who have been treated with ECU in either Period 1 or 2; *N* for Total represents the total number of patients in the Safety Set; TEAE = treatment‐emergent adverse event.

^a^ Safety set, 1 Asian patient discontinued the study before the first IP administration, thus was excluded in the Safety Set.

Percentages were based on *N* in each column.

AEs were coded to System Organ Class and Preferred Term using MedDRA, Version 21.0 coding dictionary.

Severity assessment was done in accordance with NCI‐CTCAE 5.0.

If a patient had multiple events with different severity (or causality), then the patient was counted only once at the worst severity (or causality) for the number of patients (*n*).

An event is reported under the treatment the patient was last received prior to the event.

## 4 Discussion

The pivotal Phase III cross‐over study demonstrated that SB12 is clinically equivalent to reference ECU in terms of efficacy, PK, PD, safety, and immunogenicity in complement inhibitor‐naïve patients [13]. This post‐hoc subgroup analysis explored predefined endpoints in Asian and Non‐Asian populations to assess comparability by race, despite limitations in sample size. Results showed no clinically significant differences in primary and secondary efficacy endpoints such as LDH reduction as well as additional (post‐hoc) endpoints like transfusion avoidance and hemoglobin stabilization across racial subgroups, aligning with outcomes observed in the overall population [13].

Safety outcomes for SB12 were consistent between Asians and Non‐Asian subgroups, with no unexpected adverse events and no reports of meningococcal infections during the study. These results reinforce the known safety profile of ECU [8, 9]. Clinicians should remain vigilant regarding the risk of meningococcal infection associated with complement inhibitor therapy and adhere to preventative strategies, including meningococcal vaccination and/or prophylactic antibiotic therapy before starting treatment, and patient education.

Rare genetic polymorphisms in the C5 protein have been associated with reduced responses to ECU, particularly in Japanese, Korean, and African patient populations [17, 18]. However, in this study, no patients discontinued treatment due to lack of efficacy (e.g., C5 genetic variants), as reflected in the LDH efficacy data. Subgroup analysis demonstrated no difference in the primary efficacy outcome between SB12 and ECU in the Asian and Non‐Asian patients. Despite this, Asian patients required more transfusions during the study, which may be attributed to differences in baseline characteristics. A slightly higher proportion of Asian patients had received prior pRBC transfusions (66.7% vs. 52.2%), and the mean number of pRBC units transfused in the 12 months before screening was 6.9 in Asian patients compared to 3.0 in Non‐Asian patients (Table S2). These baseline differences likely contributed to the observed transfusion requirements. Notably, LDH reduction, a key marker of hemolysis, and AUEC of LDH were comparable between Asian and Non‐Asian patients, suggesting that the higher transfusion needs in Asian patients were more likely due to baseline characteristics rather than differences in treatment efficacy.

While biosimilars like SB12 are intended to enhance accessibility to high‐cost therapies [19], it is important to acknowledge that the price differential between SB12 and reference ECU is currently modest. This limits its impact on treatment accessibility in low‐income countries, where the need for cost‐effective therapies is particularly acute. The only cure for PNH is a bone marrow transplant (BMT), but there are inherent risks associated with this procedure [3, 20]. BMT remains the best treatment option for eligible PNH patients who have no access to complement‐inhibiting treatment, as is the case in many low‐middle‐income countries [20], where common management strategies include supportive care, anticoagulants, and immunosuppressive therapies. Further efforts to reduce manufacturing and distribution costs could help address this gap and improve access to life‐saving complement inhibitor therapy.

Despite these considerations, the findings of this Phase III post‐hoc analysis support the comparable efficacy and safety of SB12 and ECU across racial subgroups, consistent with the robust data from the Phase III study [13]. SB12 has been approved by multiple regulatory authorities worldwide, including the EMA (May 2023), the United Kingdom's Medicines and Healthcare products Regulatory Agency (MHRA, October 2023), Korea's Ministry of Food and Drug Safety (MFDS, January 2024), and US Food and Drug Administration (FDA, July 2024), underscoring the rigorous standards met during its development. Biosimilars like EPYSQLI (SB12) expand treatment options and represent an important step toward addressing high costs associated with reference biologics, improving access and adherence to lifesaving therapies for PNH.

## 5 Conclusions

In summary, this post‐hoc analysis of Phase III pivotal study results by subgroup (race) in Asians and Non‐Asians supports the clinical efficacy of SB12 and its comparability with that of ECU in complement inhibitor‐naïve PNH patients, by measuring LDH (primary and secondary efficacy endpoints), units of pRBCs transfused (secondary efficacy endpoints) and two newly analyzed (post‐hoc) efficacy endpoints of transfusion avoidance and hemoglobin stabilization. The safety profiles were also comparable between SB12 and ECU‐treated patients by race subgroup (Asians and Non‐Asians), supporting the clinical use of SB12 for the treatment of PNH patients globally.

## Author Contributions

Régis Peffault de Latour, Jun Ho Jang, Jihye Park, Younsoo Kim, and Paola Russo contributed to the conceptualization of the study. Jun Ho Jang, Jihye Park, Younsoo Kim, Jinah Jung, Paola Russo, and Régis Peffault de Latour were responsible for the methodology. Younsoo Kim performed the formal analysis. Jun Ho Jang, Ciprian Tomuleasa, Hanna Oliynyk, Theerin Lanamtieng, and Soo Min Lim engaged in the clinical trial as an investigator. Data curation was performed by Jun Ho Jang, Jinah Jung, and Paola Russo. Jun Ho Jang supervised the project. All authors contributed to writing, reviewing, and editing the manuscript and have read and approved the final version.

## Conflicts of Interest

Jun Ho Jang, Ciprian Tomuleasa, Hanna Oliynyk, Theerin Lanamtieng, Soo Min Lim, and Regis Reffault de Latour declare no conflicts of interest. Younsoo Kim, Jihye Park, Jinah Jung, and Paola Russo are employees of Samsung Bioepis.

## Ethics Statement

This study was conducted in compliance with the International Council for Harmonization and Good Clinical Practice guidelines and the Declaration of Helsinki. The study and clinical study protocol, informed consent forms (ICFs), and other applicable documents were reviewed and approved by an Independent Ethics Committee (IEC) or Institutional Review Board (IRB) for each study center.

## Patient Consent Statement

Informed consent was obtained from each patient before entering the study.

## Clinical Trial Registration

ClinicalTrials.gov identifier NCT04058158.

## Supporting information



Supporting Information

## Data Availability

Data are available on request from the authors.
