# Supplementary material for: EPYSQLI (SB12; Biosimilar to Reference Eculizumab) in Asian and Non‐Asian Patients With Paroxysmal Nocturnal Hemoglobinuria: Subgroup Analysis of a Global Phase III Randomized Controlled Trial
Source: EJHaem. 2025 Mar 21;6(2):e70020. doi: 10.1002/jha2.70020 (PMC11927008; doi:10.1002/jha2.70020)
Supplement: Supplementary file 1 — Supporting Information [file JHA2-6-e70020-s001.docx]

*Article*

**Title:** **EPYSQLI™ (SB12; Biosimilar to Reference Eculizumab) in Asian and Non-Asian Patients with Paroxysmal Nocturnal Hemoglobinuria: Subgroup Analysis of a Global Phase III Randomized Controlled Trial**

**Supplementary materials**

**Table 1.** Baseline Demographics Characteristics by Planned Treatment Sequence by Race (Asian/Non-Asian) (Randomised Set)

| **Asians**  **Baseline Characteristics** | **ECU to SB12 N=12** | **SB12 to ECU N=15** | **Total N=27** |
| --- | --- | --- | --- |
| Age (years) [1] |  |  |  |
| n | 12 | 15 | 27 |
| Mean | 34.0 | 36.4 | 35.3 |
| SD | 11.30 | 11.78 | 11.41 |
| Median | 33.0 | 37.0 | 34.0 |
| Min, Max | 18, 53 | 19, 63 | 18, 63 |
| Age group, n(%) |  |  |  |
| < 65 years | 12 (100.0) | 15 (100.0) | 27 (100.0) |
| >= 65 years | 0 ( 0.0) | 0 ( 0.0) | 0 ( 0.0) |
| Gender, n(%) |  |  |  |
| Male | 7 ( 58.3) | 10 ( 66.7) | 17 ( 63.0) |
| Female | 5 ( 41.7) | 5 ( 33.3) | 10 ( 37.0) |
| Child bearing potential, n(%) [2] |  |  |  |
| Yes | 4 ( 80.0) | 4 ( 80.0) | 8 ( 80.0) |
| No | 1 ( 20.0) | 1 ( 20.0) | 2 ( 20.0) |
| Race, n(%) |  |  |  |
| American Indian or Alaska Native | 0 ( 0.0) | 0 ( 0.0) | 0 ( 0.0) |
| Asian | 12 (100.0) | 15 (100.0) | 27 (100.0) |
| Black or African American | 0 ( 0.0) | 0 ( 0.0) | 0 ( 0.0) |
| Native Hawaiian or other pacific islander | 0 ( 0.0) | 0 ( 0.0) | 0 ( 0.0) |
| White | 0 ( 0.0) | 0 ( 0.0) | 0 ( 0.0) |
| Other | 0 ( 0.0) | 0 ( 0.0) | 0 ( 0.0) |
|  | | | |
| Ethnicity, n(%) |  |  |  |
| Hispanic or Latino | 0 ( 0.0) | 0 ( 0.0) | 0 ( 0.0) |
| Indian (Indian subcontinent) | 4 ( 33.3) | 2 ( 13.3) | 6 ( 22.2) |
| Chinese | 3 ( 25.0) | 5 ( 33.3) | 8 ( 29.6) |
| Japanese | 0 ( 0.0) | 0 ( 0.0) | 0 ( 0.0) |
| Mixed ethnicity | 0 ( 0.0) | 0 ( 0.0) | 0 ( 0.0) |
| Other | 5 ( 41.7) | 8 ( 53.3) | 13 ( 48.1) |
| Height at Screening (cm) |  |  |  |
| n | 12 | 15 | 27 |
| Mean | 161.92 | 165.60 | 163.96 |
| SD | 9.258 | 8.684 | 8.964 |
| Median | 162.00 | 165.00 | 164.00 |
| Min, Max | 146.0, 180.0 | 149.0, 180.0 | 146.0, 180.0 |
| Weight at Baseline (kg) |  |  |  |
| n | 12 | 15 | 27 |
| Mean | 62.23 | 60.58 | 61.31 |
| SD | 17.212 | 10.574 | 13.647 |
| Median | 58.90 | 62.00 | 59.80 |
| Min, Max | 43.0, 111.0 | 47.0, 86.5 | 43.0, 111.0 |
| Body Mass Index (BMI) (kg/m2) [3] |  |  |  |
| n | 12 | 15 | 27 |
| Mean | 23.56 | 22.02 | 22.70 |
| SD | 5.022 | 2.907 | 3.978 |
| Median | 22.05 | 21.40 | 21.40 |
| Min, Max | 18.4, 37.5 | 17.4, 29.5 | 17.4, 37.5 |

| **Non-Asians**  **Baseline Characteristics** | | **ECU to SB12 N=13** | **SB12 to ECU N=10** | **Total N=23** | |
| --- | --- | --- | --- | --- | --- |
| Age (years) [1] | |  |  |  | |
| n | | 13 | 10 | 23 | |
| Mean | | 38.4 | 45.3 | 41.4 | |
| SD | | 15.71 | 14.61 | 15.30 | |
| Median | | 38.0 | 49.5 | 38.0 | |
| Min, Max | | 19, 79 | 27, 65 | 19, 79 | |
| Age group, n(%) | |  |  |  | |
| < 65 years | | 12 ( 92.3) | 9 ( 90.0) | 21 ( 91.3) | |
| >= 65 years | | 1 ( 7.7) | 1 ( 10.0) | 2 ( 8.7) | |
| Gender, n(%) | |  |  |  | |
| Male | | 4 ( 30.8) | 7 ( 70.0) | 11 ( 47.8) | |
| Female | | 9 ( 69.2) | 3 ( 30.0) | 12 ( 52.2) | |
| Child bearing potential, n(%) [2] | |  |  |  | |
| Yes | | 8 ( 88.9) | 2 ( 66.7) | 10 ( 83.3) | |
| No | | 1 ( 11.1) | 1 ( 33.3) | 2 ( 16.7) | |
| Race, n(%) | |  |  |  | |
| American Indian or Alaska Native | | 0 ( 0.0) | 0 ( 0.0) | 0 ( 0.0) | |
| Asian | | 0 ( 0.0) | 0 ( 0.0) | 0 ( 0.0) | |
| Black or African American | | 0 ( 0.0) | 0 ( 0.0) | 0 ( 0.0) | |
| Native Hawaiian or other pacific islander | | 0 ( 0.0) | 0 ( 0.0) | 0 ( 0.0) | |
| White | | 11 ( 84.6) | 7 ( 70.0) | 18 ( 78.3) | |
| Other | | 2 ( 15.4) | 3 ( 30.0) | 5 ( 21.7) | |
| Ethnicity, n(%) | |  |  |  | |
| Hispanic or Latino | | 2 ( 15.4) | 3 ( 30.0) | 5 ( 21.7) | |
| Indian (Indian subcontinent) | | 0 ( 0.0) | 0 ( 0.0) | 0 ( 0.0) | |
| Chinese | | 0 ( 0.0) | 0 ( 0.0) | 0 ( 0.0) | |
| Japanese | | 0 ( 0.0) | 0 ( 0.0) | 0 ( 0.0) | |
| Mixed ethnicity | | 0 ( 0.0) | 0 ( 0.0) | 0 ( 0.0) | |
| Other | | 11 ( 84.6) | 7 ( 70.0) | 18 ( 78.3) | |
|  | |  |  |  | |
| Height at Screening (cm) |  | |  | |  |
| n | 13 | | 10 | | 23 |
| Mean | 166.15 | | 170.50 | | 168.04 |
| SD | 7.448 | | 11.068 | | 9.232 |
| Median | 163.00 | | 170.50 | | 170.00 |
| Min, Max | 155.0, 178.0 | | 151.0, 190.0 | | 151.0, 190.0 |
| Weight at Baseline (kg) |  | |  | |  |
| n | 13 | | 10 | | 23 |
| Mean | 66.89 | | 80.22 | | 72.69 |
| SD | 14.661 | | 12.681 | | 15.122 |
| Median | 61.00 | | 81.00 | | 72.00 |
| Min, Max | 43.0, 94.1 | | 57.0, 100.0 | | 43.0, 100.0 |
| Body Mass Index (BMI) (kg/m2) [3] |  | |  | |  |
| n | 13 | | 10 | | 23 |
| Mean | 24.03 | | 27.47 | | 25.53 |
| SD | 3.779 | | 2.378 | | 3.625 |
| Median | 23.00 | | 27.35 | | 25.80 |
| Min, Max | 16.8, 29.7 | | 24.3, 31.1 | | 16.8, 31.1 |

- Percentages were based on the number of subjects in the Randomized Set, unless otherwise specified.

- [1] Age was calculated as Age (years)= year of informed consent - year of birth.

- [2] Percentages were based on the number of female subjects.

- [3] BMI = (body weight in kilograms)/(height in meters)^2

**Table 2.** Summary Statistics for Disease-related Laboratory Parameters by Planned Treatment Sequence (Modified Full Analysis Set) and by Race (Asian/Non-Asian)

| **Asian**  **Disease Characteristics** | **ECU to SB12 N=12** | | **SB12 to ECU N=15** | | **Total N=27** | |
| --- | --- | --- | --- | --- | --- | --- |
| Duration of PNH (years) [1] |  | |  | |  | |
| n | 12 | | 15 | | 27 | |
| Mean | 4.043 | | 4.862 | | 4.498 | |
| SD | 4.1537 | | 3.8870 | | 3.9506 | |
| Median | 1.870 | | 2.930 | | 2.400 | |
| Min, Max | 0.02, 11.70 | | 0.65, 13.04 | | 0.02, 13.04 | |
| Subject received a prior pRBC transfusion, n(%) |  | |  | |  | |
| Yes | 9 ( 75.0) | | 9 ( 60.0) | | 18 ( 66.7) | |
| No | 3 ( 25.0) | | 6 ( 40.0) | | 9 ( 33.3) | |
| Total number of pRBCs in 12 months prior to Screening [2] |  | |  | |  | |
| n | 12 | | 15 | | 27 | |
| Mean | 5.0 | | 8.3 | | 6.9 | |
| SD | 4.43 | | 9.12 | | 7.48 | |
| Median | 5.5 | | 4.0 | | 5.0 | |
| Min, Max | 0, 12 | | 0, 28 | | 0, 28 | |
| LDH (U/L) at Baseline | |  | |  | |  |
| n | | 12 | | 15 | | 27 |
| Mean | | 2736.9 | | 2258.4 | | 2471.1 |
| SD | | 1659.91 | | 939.88 | | 1303.87 |
| Median | | 2302.5 | | 2235.0 | | 2235.0 |
| Min, Max | | 660, 6630 | | 678, 4245 | | 660, 6630 |
| PNH clone size of Type II RBCs (%) at Baseline | |  | |  | |  |
| n | | 12 | | 15 | | 27 |
| Mean | | 5.855 | | 13.576 | | 10.144 |
| SD | | 6.8630 | | 22.5125 | | 17.5531 |
| Median | | 2.595 | | 3.380 | | 3.370 |
| Min, Max | | 0.15, 21.48 | | 0.07, 66.27 | | 0.07, 66.27 |
| PNH clone size of Type III RBCs (%) at Baseline | |  | |  | |  |
| n | | 12 | | 15 | | 27 |
| Mean | | 39.735 | | 27.290 | | 32.821 |
| SD | | 24.9019 | | 17.9976 | | 21.8284 |
| Median | | 34.520 | | 22.210 | | 30.120 |
| Min, Max | | 7.42, 92.05 | | 4.25, 58.71 | | 4.25, 92.05 |
| Total PNH clone size of RBCs (%) at Baseline | |  | |  | |  |
| n | | 12 | | 15 | | 27 |
| Mean | | 45.590 | | 40.866 | | 42.966 |
| SD | | 24.5242 | | 25.8418 | | 24.8950 |
| Median | | 48.410 | | 36.360 | | 42.790 |
| Min, Max | | 8.92, 92.53 | | 4.92, 84.66 | | 4.92, 92.53 |
| PNH clone size of Granulocytes (%) at Baseline | |  | |  | |  |
| n | | 12 | | 15 | | 27 |
| Mean | | 92.141 | | 88.729 | | 90.246 |
| SD | | 8.2893 | | 23.8750 | | 18.4116 |
| Median | | 96.185 | | 94.070 | | 94.860 |
| Min, Max | | 76.44, 99.57 | | 3.33, 99.68 | | 3.33, 99.68 |
| PNH clone size of Monocytes (%) at Baseline | |  | |  | |  |
| n | | 12 | | 15 | | 27 |
| Mean | | 93.818 | | 93.322 | | 93.542 |
| SD | | 6.1566 | | 7.1961 | | 6.6319 |
| Median | | 95.680 | | 96.890 | | 95.820 |
| Min, Max | | 78.11, 98.56 | | 71.74, 99.55 | | 71.74, 99.55 |
| Reticulocyte count (%) at Baseline | |  | |  | |  |
| n | | 12 | | 15 | | 27 |
| Mean | | 9.714 | | 9.001 | | 9.318 |
| SD | | 5.8077 | | 3.8856 | | 4.7466 |
| Median | | 7.235 | | 9.920 | | 9.310 |
| Min, Max | | 3.70, 21.60 | | 3.32, 15.00 | | 3.32, 21.60 |
| Haptoglobin (g/L) at Baseline | |  | |  | |  |
| n | | 12 | | 15 | | 27 |
| Mean | | 0.050 | | 0.050 | | 0.050 |
| SD | | 0.0000 | | 0.0000 | | 0.0000 |
| Median | | 0.050 | | 0.050 | | 0.050 |
| Min, Max | | 0.05, 0.05 | | 0.05, 0.05 | | 0.05, 0.05 |
| Haemoglobin (g/L) at Baseline | |  | |  | |  |
| n | | 12 | | 15 | | 27 |
| Mean | | 80.3 | | 86.7 | | 83.9 |
| SD | | 17.92 | | 28.37 | | 24.07 |
| Median | | 81.5 | | 88.0 | | 83.0 |
| Min, Max | | 42, 104 | | 38, 136 | | 38, 136 |
|  | | | | | | |

| **Non-Asian**  **Disease Characteristics** | **ECU to SB12 N=13** | **SB12 to ECU N=10** | **Total N=23** |
| --- | --- | --- | --- |
| Duration of PNH (years) [1] |  |  |  |
| n | 13 | 10 | 23 |
| Mean | 6.008 | 10.953 | 8.158 |
| SD | 7.7154 | 10.2670 | 9.0484 |
| Median | 4.050 | 8.070 | 4.570 |
| Min, Max | 0.00, 22.56 | 0.14, 29.28 | 0.00, 29.28 |
| Subject received a prior pRBC transfusion, n(%) |  |  |  |
| Yes | 5 ( 38.5) | 7 ( 70.0) | 12 ( 52.2) |
| No | 8 ( 61.5) | 3 ( 30.0) | 11 ( 47.8) |
| Total number of pRBCs in 12 months prior to Screening [2] |  |  |  |
| n | 13 | 10 | 23 |
| Mean | 2.6 | 3.4 | 3.0 |
| SD | 5.11 | 4.06 | 4.60 |
| Median | 0.0 | 2.0 | 1.0 |
| Min, Max | 0, 18 | 0, 11 | 0, 18 |
| LDH (U/L) at Baseline |  |  |  |
| n | 13 | 10 | 23 |
| Mean | 1619.8 | 2162.8 | 1855.9 |
| SD | 990.57 | 1259.19 | 1122.33 |
| Median | 1275.0 | 1897.5 | 1680.0 |
| Min, Max | 546, 3840 | 872, 5430 | 546, 5430 |
| PNH clone size of Type II RBCs (%) at Baseline |  |  |  |
| n | 12 | 10 | 22 |
| Mean | 9.333 | 20.297 | 14.316 |
| SD | 15.5064 | 30.3829 | 23.5116 |
| Median | 1.970 | 5.825 | 3.605 |
| Min, Max | 0.46, 54.68 | 0.07, 88.03 | 0.07, 88.03 |
| PNH clone size of Type III RBCs (%) at Baseline |  |  |  |
| n | 13 | 10 | 23 |
| Mean | 44.853 | 40.809 | 43.095 |
| SD | 22.4641 | 21.8552 | 21.7913 |
| Median | 43.260 | 40.500 | 43.230 |
| Min, Max | 1.99, 96.92 | 6.58, 74.48 | 1.99, 96.92 |
| Total PNH clone size of RBCs (%) at Baseline |  |  |  |
| n | 13 | 10 | 23 |
| Mean | 53.468 | 59.477 | 56.080 |
| SD | 26.6938 | 23.6028 | 25.0169 |
| Median | 55.600 | 63.230 | 59.260 |
| Min, Max | 9.58, 99.62 | 27.87, 94.61 | 9.58, 99.62 |
| PNH clone size of Granulocytes (%) at Baseline |  |  |  |
| n | 13 | 10 | 23 |
| Mean | 85.445 | 91.457 | 88.059 |
| SD | 15.6512 | 7.2678 | 12.8261 |
| Median | 93.440 | 90.975 | 91.400 |
| Min, Max | 54.77, 99.73 | 74.14, 98.39 | 54.77, 99.73 |
| PNH clone size of Monocytes (%) at Baseline |  |  |  |
| n | 13 | 10 | 23 |
| Mean | 85.562 | 91.750 | 88.252 |
| SD | 15.2074 | 8.3977 | 12.8387 |
| Median | 94.880 | 94.860 | 94.880 |
| Min, Max | 59.08, 99.78 | 72.94, 99.10 | 59.08, 99.78 |
| Reticulocyte count (%) at Baseline |  |  |  |
| n | 13 | 10 | 23 |
| Mean | 6.212 | 7.733 | 6.873 |
| SD | 4.1940 | 3.6540 | 3.9561 |
| Median | 5.300 | 8.415 | 5.320 |
| Min, Max | 1.57, 15.39 | 2.17, 13.96 | 1.57, 15.39 |
| Haptoglobin (g/L) at Baseline |  |  |  |
| n | 13 | 10 | 23 |
| Mean | 0.050 | 0.050 | 0.050 |
| SD | 0.0000 | 0.0000 | 0.0000 |
| Median | 0.050 | 0.050 | 0.050 |
| Min, Max | 0.05, 0.05 | 0.05, 0.05 | 0.05, 0.05 |
| Haemoglobin (g/L) at Baseline |  |  |  |
| n | 13 | 10 | 23 |
| Mean | 91.0 | 92.8 | 91.8 |
| SD | 21.06 | 19.88 | 20.11 |
| Median | 93.0 | 90.0 | 91.0 |
| Min, Max | 57, 121 | 73, 145 | 57, 145 |
|  | | | |

- PNH: Paroxysmal Nocturnal Haemoglobinuria; pRBC: Packed Red Blood Cells; LDH: Lactate dehydrogenase; RBC: Red Blood Cell; WBC: White Blood Cell; SD: Standard Deivation.

- Percentages were based on the number of subjects in the Randomised Set, unless otherwise specified.

- Total PNH clone size of RBC is calculated as the sum of PNH clone size of type II and type III RBC. When PNH clone size of type II RBC is missing, only PNH clone size of type III RBC was used to calculated Total PNH clone size of RBC.

- [1] Duration of PNH (years) was calculated as the (date of informed consent - diagnosed date of PNH +1)/365.25.

- [2] For subjects who didn't have prior pRBCs, the total number of pRBCs in 12 months prior to Screening was set to 0.
